# A Pseudovirus‐Based Method for the Simultaneous Quantitation of Neutralization Antibodies Against All Three Poliovirus Serotypes

**DOI:** 10.1002/mco2.70551

**Published:** 2025-12-17

**Authors:** Meiyan Liu, Yuanling Yu, Yadong Li, Zexin Tao, Lan Huang, Xi Wu, Yong Zhang, Shuangli Zhu, Qiang Sun, Tianjiao Ji, Dongyan Wang, Ziteng Liang, Shuo Liu, Meina Cai, Yimeng An, Jierui Li, Weijin Huang, Guoyang Liao, Li Yi, Lei Ma, Li Zhang, Youchun Wang

**Affiliations:** ^1^ Chinese Academy of Medical Sciences & Peking Union Medical College Beijing China; ^2^ Changping Laboratory Beijing China; ^3^ Institute of Medical Biology Chinese Academy of Medical Sciences Kunming China; ^4^ Shandong Center for Disease Control and Prevention Jinan China; ^5^ Division of HIV/AIDS and Sexually‐transmitted Virus Vaccines Institute for Biological Product Control National Institutes for Food and Drug Control (NIFDC) Beijing China; ^6^ National Key Laboratory of Intelligent Tracking and Forecasting for Infectious Diseases (NITFID) National Institute for Viral Disease Control and Prevention Chinese Center for Disease Control and Prevention Beijing China; ^7^ National Polio Laboratory World Health Organization Polio Reference Laboratory for the Western Pacific Region National Institute for Viral Disease Control and Prevention Chinese Center for Disease Control and Prevention Beijing China; ^8^ National Health Commission Key Laboratory of Laboratory Biosafety National Institute for Viral Disease Control and Prevention Chinese Center for Disease Control and Prevention Beijing China; ^9^ School of Public Health Shandong University Jinan China; ^10^ Immunization Program Management Office Shandong Provincial Center for Disease Control and Prevention Jinan China

**Keywords:** poliovirus, fluorescent pseudovirus, simultaneous quantitation, neutralizing antibodies

## Abstract

Poliovirus is characterized by three antigenically distinct serotypes that do not elicit cross‐neutralizing antibodies. In the final stages of poliovirus eradication, the gold‐standard conventional neutralization test (cNT) for detecting serum neutralizing antibodies (NAbs) is highly restricted due to biosafety concerns. To address this, we developed a high‐throughput, tri‐color pseudovirus‐based neutralization assay (PBNA) for the simultaneous quantification of NAbs against all three poliovirus serotypes. We generated pseudoviruses by co‐transfecting cells with P1 plasmids, a replication plasmid, and a T7 RNA polymerase plasmid. By optimizing P1 expression, sensitive cell selection (HEK 293T), and plasmid transfection ratios (3:3:1 for P1, replicon, and T7 plasmids), we produced high‐titer pseudoviruses (>29‐fold increase in titers). Based on high‐titer pseudovirus encoding distinct fluorophores (E2, eGFP, and RFP), the PBNA was established, which was optimized for a 12 h incubation period, 4 × 10⁴ cells per well, and 1500 TCID_50_/mL of pseudovirus. It demonstrated high sensitivity, strong serotype specificity, and excellent reproducibility. Furthermore, the PBNA and cNT exhibited excellent congruency (*r* > 0.88, all serotypes). The tri‐color PBNA provides a safe, rapid, and alternative to the cNT, making it an invaluable tool for large‐scale serosurveillance, novel vaccine evaluation, and fundamental virological investigations in the post‐eradication era.

## Graphical abstract

By optimizing P1 expression, sensitive cell selection, and plasmid transfection ratios, we produced a high‐titer poliovirus pseudovirus. Based on high‐titer pseudovirus encoding distinct fluorophores (E2, eGFP, and RFP), we developed a tri‐color pseudovirus‐based neutralization assay (PBNA) for the simultaneous quantification of neutralizing antibodies (NAbs) against all three poliovirus serotypes, which shows excellent congruency with the conventional neutralization test (cNT). The method demonstrated high sensitivity, strong serotype specificity, and excellent reproducibility.

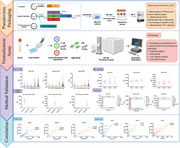

## 1 Introduction

Poliomyelitis (polio) is an acute intestinal infection caused by poliovirus (PV), which mainly affects children under five years of age [1]. Poliovirus is primarily transmitted through the fecal–oral route, but it can also invade the central nervous system, which can lead to irreversible limb paralysis in severe cases [2, 3]. Due to the remarkable success of the Global Polio Eradication Initiative (GPEI), wild poliovirus (WPV) types 2 and 3 have been declared eradicated, with only WPV1 remaining in Afghanistan and Pakistan [4, 5]. As humanity draws closer to the goal of completely eradicating poliovirus, accurately assessing population immunity levels and monitoring novel vaccine efficacy have become critical.

Poliovirus, belonging to the Enterovirus genus in the family Picornaviridae, has a virion with a diameter of 20–30 nm without an envelope, containing a single‐stranded positive‐sense RNA genome [6, 7]. The length of the RNA genome of poliovirus is approximately 7500 nt [8], and it is mainly divided into three parts: the noncoding region at the 5'‐end, the polyprotein coding region, and the noncoding region at the 3'‐end. The polyprotein coding region is subdivided into three parts: P1, P2, and P3 [9]. The P1 region encodes a structural protein with the major antigenic determinants VP4, VP2, VP3, and VP1. The P2 and P3 regions encode nonstructural proteins, which regulate proteolytic processing and replication [10, 11]. Based on antigenic properties, polioviruses are classified into three serotypes, with no cross‐immunity between them [7].

The detection of neutralizing antibodies (NAbs) against poliovirus is important for evaluating the effectiveness of polio vaccination, diagnosing poliovirus infection, monitoring transmission, and formulating public health strategies. The conventional neutralization test (cNT) is the gold standard for serological assessment of poliovirus NAbs [12]. However, due to strict restrictions on the use of infectious authentic poliovirus after its containment, the gold standard cNT method is operationally unsuitable for such large‐scale surveillance tasks. Furthermore, the cNT makes the process labor‐intensive, time‐consuming, and dependent on subjective human interpretation of the cytopathic effect (CPE). Most importantly, it has high biosafety requirements, necessitating performance in designated Biosafety Level 3 (BSL‐3) laboratories. These limitations, coupled with the ongoing global effort to eradicate polio and the poliovirus sequestration program, have severely restricted the use of authentic poliovirus, creating substantial challenges for fundamental research on poliovirus pathogenesis, as well as for population immunity monitoring and post‐eradication vaccine efficacy evaluation. Given these constraints, there is an urgent need for alternative methods for detecting poliovirus that do not require the use of infectious live virus. While enzyme‐linked immunosorbent assays (ELISA) offer a solution by eliminating the need for infectious poliovirus and reducing assay time, they suffer from limitations such as low sensitivity, narrow linearity, weak correlation with authentic virus neutralization assays, and a focus on spatial epitopes that do not adequately reflect NAbs function [13, 14, 15, 16]. The pseudovirus‐based neutralization assay (PBNA) provides a promising alternative to traditional neutralization assays [17, 18, 19, 20, 21, 22, 23, 24, 25, 26]. This approach eliminates the need for infectious authentic viruses, offers enhanced safety (can be performed in a BSL‐2 laboratory), reduces testing time, and allows for objective quantification. However, current PBNA methodologies typically employ pseudoviruses with reporter genes that require chemical reagents, such as luciferase, and enable the detection of only one NAbs per assay, limiting throughput [26, 27, 28, 29, 30, 31]. Additionally, chemiluminescence‐based detection methods are expensive and necessitate exogenous substrates.

Therefore, to address the critical problems left by the biocontainment restrictions on the cNT and the functional or throughput limitations of existing alternatives, we developed a high‐throughput trivalent fluorescent PBNA capable of simultaneously detecting NAbs against all three poliovirus serotypes. This assay, based on a 96‐well plate format, combines the advantages of high throughput, automation, low sample amount, cost‐effectiveness, and user‐friendliness, providing a robust tool for large‐scale monitoring of NAbs and the evaluation of vaccine efficacy.

## 2 Results

### 2.1 Preparation of High‐Titer Fluorescent Poliovirus Pseudovirus

#### 2.1.1 Optimization of Fluorescent Protein Reporter Genes for Different Poliovirus Serotypes

**FIGURE 1:**
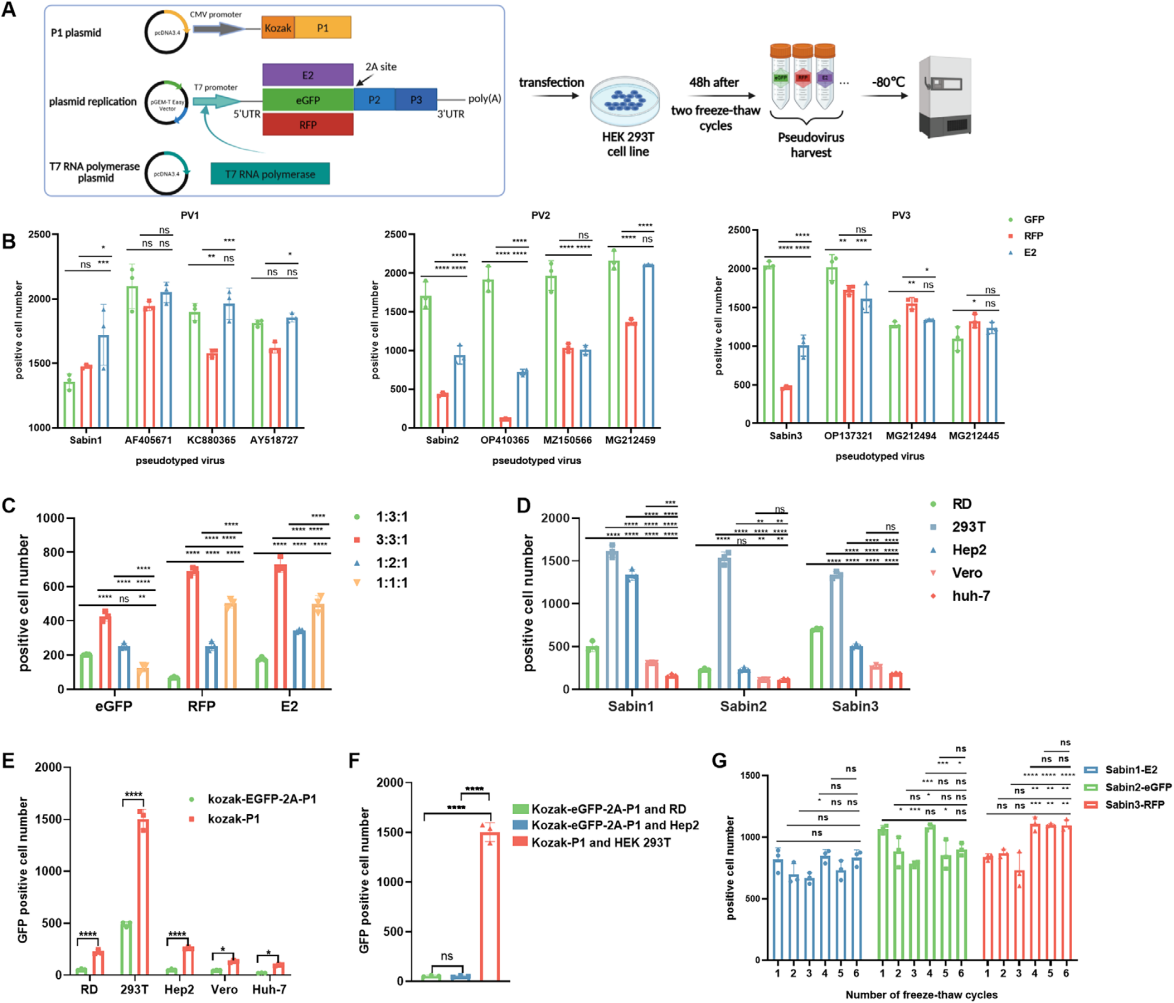
Preparation and optimization of fluorescent poliovirus pseudovirus. (A) Schematic diagram of the three‐plasmid pseudovirus system and the procedure of pseudovirus production. Pseudoviruses were obtained by co‐transfection of packaging cells with three plasmids. P1 plasmid encoding the specific capsid proteins from a specific poliovirus strain. Replicon plasmid that serves as a common backbone, providing the nonstructural proteins and a fluorescent reporter gene; T7 RNA polymerase plasmid drives the transcription of the replicon. (B) Optimization of the selection of fluorescent protein reporter genes for different serotypes of poliovirus pseudovirus. Pseudoviruses corresponding to four strains each of type 1, 2, and 3 were prepared using a replicon plasmid containing three different fluorescent protein genes (eGFP, RFP, and E2) for a total of 12 strains and 36 combinations. The constructed poliovirus pseudoviruses included Sabin strains as well as nine randomly selected strains from NCBI, with GenBank accession numbers AY184219 (Sabin1), AF405671, KC880365, AY518727, and for type 1; AY184220 (Sabin2), OP410365, MZ150566, and MG212459 type 2; as well as AY184221 (Sabin3), OP137321, MG212494, and MG212445 for type 3. (C) Optimization of transfection ratios of the three plasmids. Cells were transfected with the above three plasmids in different ratios (1:3:1, 3:3:1, 1:2:1, and 1:1:1). The effects of the different transfection ratios of the three plasmids on the fluorescent pseudovirus titers were compared. (D) Selection of sensitive cell lines. To select the best sensitive cell line for the assay, we tested the sensitivity of RD, HEK 293T, Hep2, Vero, and Huh‐7 cells to poliovirus pseudovirus. (E) Optimized P1 plasmid increased the pseudovirus titer. The eGFP‐positive cell number following pseudovirus infection was detected in RD, HEK 293T, Hep2, Vero, and Huh‐7 cells before and after optimization of the P1 plasmid. (F) Comparison of titers of pseudoviruses prepared using the optimized P1 plasmid in HEK 293T, RD, and Hep2 cells. The combination of sensitive HEK 293T cells with the Kozak‐P1 plasmid resulted in a 27.8‐fold enhancement of pseudovirus titer compared with RD and Kozak‐eGFP‐2A‐P1, while a 29.5‐fold enhancement of pseudovirus titer was observed compared with Hep2 cells combined with the Kozak‐eGFP‐2A‐P1 plasmid. Error bars indicate the standard deviation of three independent replicate tests. (G) Effect of repeated freeze‐thaw on the titers of poliovirus pseudotyped viruses. To assess stability, the number of positive HEK 293T cells was quantified after six freeze‐thaw cycles of the Sabin 1, 2, and 3 pseudoviruses. The experimental data were obtained from three repeated trials. Results are expressed as mean ± standard deviation (SD). Statistical analysis was performed using one or two‐way ANOVA followed by Tukey's multiple comparisons test. Significance levels are indicated with asterisks: *p < 0.05, **p < 0.01, ***p < 0.005, ****p < 0.001.

The P1 plasmid, replicon plasmid, and T7 RNA polymerase plasmid were used to co‐transfect for packaging the poliovirus pseudotyped viruses (Figure 1A). To screen for fluorescent protein genes, first, we used a fluorescent spot counter to detect the titers of pseudoviruses carrying reporter genes encoding nine different fluorescent proteins (eGFP, ZsGreen, YPet, RFP, mOrange, dTomato, mCherry, mKate2, and E2) in the (EX 469/35, EM 525/39), (EX 531/40, EM 593/40) and (EX 628/40, EM 685/40) bands, respectively. As shown in Table S1, we discarded YPet, mKate2, and mCherry because they interfered in the (EX 469/35, EM 525/39) band, (EX 531/40, EM 593/40) band, and EX 628/40, EM 685/40) band. Second, among the remaining six fluorescent protein genes, there were more positive cells for eGFP compared with ZsGreen in the (EX 469/35, EM 525/39) band, as well as more positive cells for RFP compared with mOrange and dTomato in the (EX 531/40, EM 593/40) band. Only E2 was unaffected by the other two excitation wavelengths and had a higher positive cell number in the (EX 628/40, EM 685/40) band. Therefore, the three fluorescent protein reporter genes eGFP, RFP, and E2 were selected. Finally, to optimize the selection of fluorescent protein reporter genes for different serotypes of poliovirus and to determine the best combinations for serotypes 1, 2, and 3, we prepared four strains of each serotype for the 36 combinations of pseudovirus of serotypes 1, 2 and 3 using replicon plasmids containing three different fluorescent protein reporter genes (eGFP, RFP, and E2). We constructed 12 poliovirus pseudovirus strains by codon‐optimizing and individually cloning the capsid (P1) coding regions from 12 distinct strains into a capsid expression plasmid. These comprised the 3 Sabin vaccine strains and 9 additional strains which were randomly selected from NCBI, with the following GenBank accession codes: for serotype 1, AY184219 (Sabin 1), AF405671, KC880365, and AY518727; for serotype 2, AY184220 (Sabin 2), OP410365, MZ150566, and MG212459; and for serotype 3, AY184221 (Sabin 3), OP137321, MG212494, and MG212445. When poliovirus serotypes 1, 2, and 3 corresponded to E2, eGFP, and RFP, the positive cell number was higher. The optimal fluorescent protein reporter genes for poliovirus serotypes 1, 2, and 3 were therefore determined to be type 1‐E2, type 2‐eGFP, and type 3‐RFP (Figure 1B).

#### 2.1.2 Optimization of the Transfection Ratio of the Three Plasmids

To package a high titer of poliovirus fluorescent pseudovirus, we co‐transfected the host cells with the P1 plasmid, replicon plasmid, and T7 RNA polymerase plasmid at different ratios (1:3:1, 3:3:1, 1:2:1, 1:1:1). The total amount of plasmid DNA was 7 µg, and the transfections were carried out in a six‐well plate. The positive cell number was highest when the transfection ratio of the above three plasmids was 3:3:1. Therefore, the optimal transfection ratio of the P1 plasmid, the replicon plasmid, and the T7 RNA polymerase plasmid was determined to be 3:3:1 (Figure 1C).

#### 2.1.3 Screening of Sensitive Cell Lines and Optimization of P1 Plasmid

To identify sensitive cell lines for use in poliovirus neutralization antibody detection, we further investigated the infectivity of Sabin1‐E2, Sabin2‐eGFP, and Sabin3‐RFP pseudoviruses in RD, HEK 293T, Hep2, Vero, and Huh‐7 cells. The positive cell numbers were all highest in HEK 293T cells, followed by RD and Hep2. Therefore, HEK 293T cells were selected for the assay (Figure 1D). To ensure the authenticity of the HEK 293T cell line, we confirmed its identity through short tandem repeat (STR) analysis (MicroreaderTM21 ID System), performed by Beijing Yueweiyin Gene Technology Co., Ltd. (Beijing, China).

The original design strategy of the P1 plasmid was Kozak‐eGFP‐2A‐P1 [31], and in this study, we found that the direct incorporation of Kozak‐P1 into the vector could enhance the pseudovirus titers in different cell lines. The pseudovirus titer of Kozak‐P1 compared with the design strategy of Kozak‐eGFP‐2A‐P1 was increased in RD, HEK 293T, Hep2, Vero, and Huh‐7 cell lines by 4.1, 3.1, 5.0, 2.9, and 4.8‐fold, respectively (Figure 1E).

To further enhance the titer of poliovirus pseudovirus, we combined the optimized capsid expression plasmid Kozak‐P1 with the highly sensitive HEK 293T cells identified in this study and compared the titers with those obtained using previously reported strategies. As shown in Figure 1F, the combination of Kozak‐P1 and HEK 293T cells yielded a 27.8‐fold increase in pseudovirus titer compared with the Kozak‐eGFP‐2A‐P1 plasmid and RD cells commonly used in chemiluminescence‐based neutralization assays (Figure 1F). Moreover, a 29.5‐fold increase was observed compared with the combination of Kozak‐eGFP‐2A‐P1 and Hep2 cells (Figure 1F). These results demonstrated that the dual optimization of both the expression plasmid and the target cell line can significantly enhance the yield of poliovirus pseudovirus. Additionally, to assess the pseudovirus stability upon storage, we subjected aliquots of Sabin 1, 2, and 3 pseudoviruses to six repeated freeze‐thaw cycles. As shown in Figure 1G, the titers of all three serotypes remained highly stable, exhibiting no significant decrease even after six cycles.

In summary, this study determined that pseudotyped poliovirus serotypes 1, 2, and 3 had the highest titers when using the E2, eGFP, and RFP fluorescent protein reporter genes, respectively, and determined that the optimal transfection ratio for the P1 plasmid, the replicon plasmid, and the T7 RNA polymerase plasmid was 3:3:1. In addition, high‐titer and stable poliovirus fluorescent pseudovirus was successfully prepared by optimizing the P1 plasmid combined with highly sensitive HEK 293T cells.

### 2.2 Development of a High‐Throughput Assay for Simultaneous Detection of NAbs Against All Three Poliovirus Serotypes

In order to establish a high‐throughput method for the detection of poliovirus NAbs, the parameters such as detection time, cell incorporation, and pseudovirus incorporation were optimized, after which the cut‐off value, specificity, and reproducibility of the assay were validated.

#### 2.2.1 Optimization of Detection Time

**FIGURE 2:**
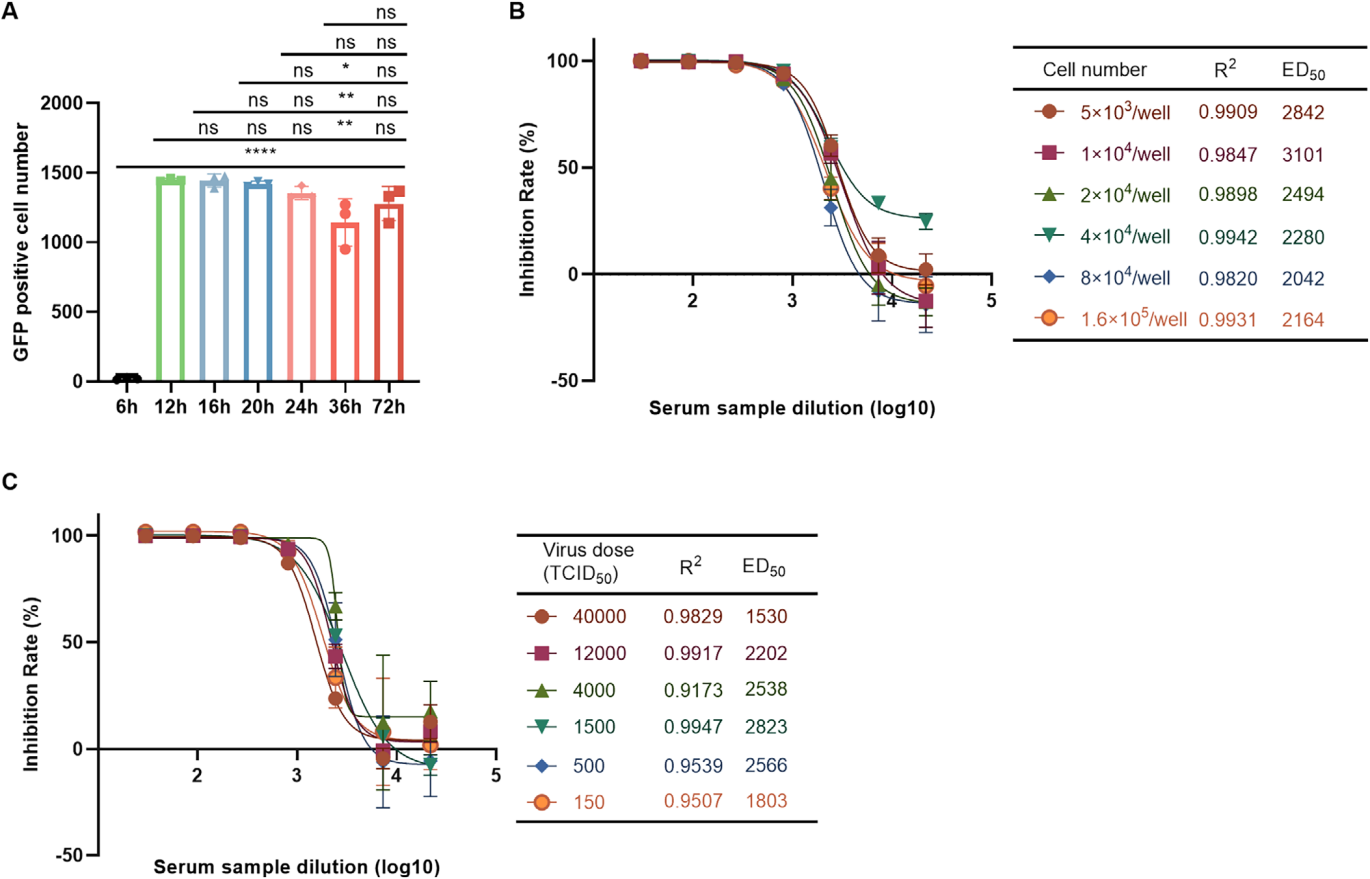
Establishment and optimization of a PBNA for the detection of NAbs against three poliovirus serotypes. (A) Selection of optimal neutralization antibody detection time. The eGFP‐positive cell numbers were determined at different time points (6, 12, 16, 20, 24, 36, and 72 h) after pseudovirus infection of HEK 293T cells. The Y‐axis indicates the number of eGFP‐positive fluorescent spots. The experimental data were obtained from three repeated trials. Results are expressed as mean ± standard deviation (SD). Statistical analysis was performed using one‐way ANOVA followed by Tukey's multiple comparisons test. Significance levels are indicated with asterisks: *p < 0.05, **p < 0.01, ***p < 0.005, ****p < 0.001. (B) Selection of the optimal amounts of sensitive cells for PBNA. The effect of cell inoculum size from 5 × 10^3^ to 1.6 × 10^5^ cells/well on the neutralization assay was assessed by calculating the ED_50_ and its corresponding *R* ^2^ value. (C) Selection of the optimal pseudovirus dose for PBNA. The effect of different pseudovirus doses ranging from 150∼40,000 TCID_50_/ml on the neutralization assay was assessed by calculating the ED_50_ and its corresponding *R* ^2^ value.

To determine the optimal detection time, we examined the number of positive cells at different times (6, 12, 16, 20, 24, 36, and 72 h) after Sabin2‐eGFP pseudovirus infection of HEK 293T cells. The results showed that the positive cell number reached the maximum value after 12 h and stabilized thereafter. Therefore, the optimal detection time was 12 h after infection (Figure 2A).

#### 2.2.2 Optimization of Cell Inoculum Size

To determine the optimal amount of sensitive cells HEK 293T, we used rat serum after immunization with a monovalent bulk of Sabin‐IPV (type 2) and Sabin2‐eGFP pseudovirus for the PBNA. The amount of HEK 293T cells was varied at 5×10^3^, 1 × 10^4^, 2 × 10^4^, 4 × 10^4^, 8 × 10^4^, and 1.6 × 10^5^ cells/well, respectively. As shown in Figure 2B, the linear correlation coefficients *R* ^2^ > 0.98 for different cell inoculum sizes suggested that the linear curves were well fitted. In addition, the ED_50_ values were more consistent at different cell amounts. Since the highest correlation coefficient (*R* ^2^ = 0.9942) was observed at 4 × 10^4^ cells/well, this was selected as the optimal cell inoculum size (Figure 2B).

#### 2.2.3 Optimization of Pseudovirus Infection

To optimize the pseudovirus infection conditions in the neutralization assay, we used rat serum after immunization with a monovalent bulk of Sabin‐IPV (type 2) and Sabin2‐eGFP pseudovirus. The steps were the same as in the PBNA, and the only difference was that the virion dose was varied at 40,000, 12,000, 4000, 1500, 500, and 150 TCID_50_/mL, respectively. As shown in Figure 2C, the linear correlation coefficients of different infectious doses were *R* ^2^ > 0.91, suggesting that the linear curves were well‐fitted. In addition, the ED_50_ values with different virion doses were comparable. Since the highest correlation coefficient (*R* ^2^ = 0.9947) was found at 1500 TCID_50_/mL, this was selected as the optimal virion dose (Figure 2C).

#### 2.2.4 Cut‐Off Value Determination for the PBNA

**FIGURE 3:**
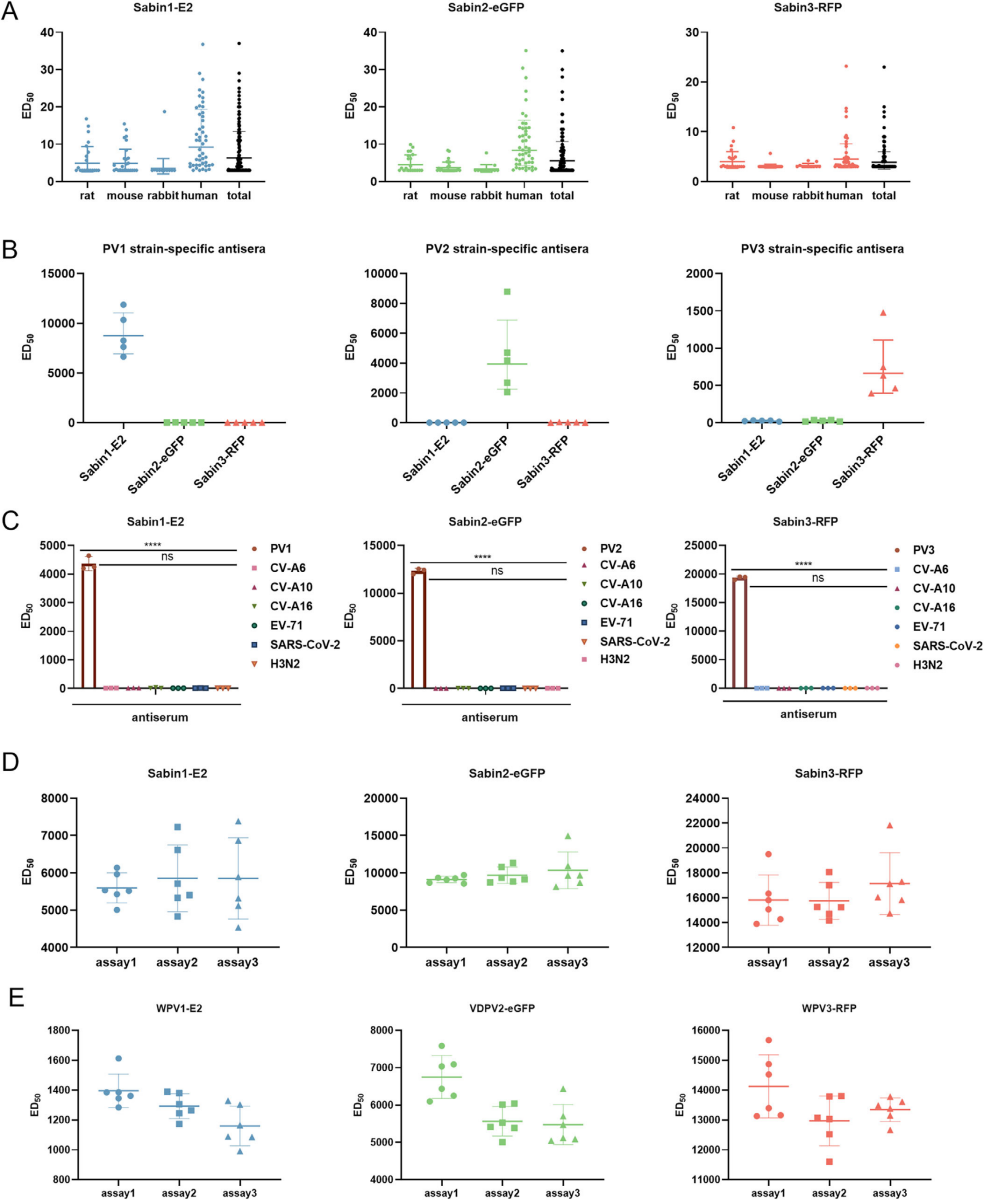
Validation of the PBNA. (A) Cut off value. The cut‐off value of the PBNA using Sabin1‐E2, Sabin2‐eGFP, and Sabin3‐RFP pseudoviruses combined with negative sera from rats (*n* = 20), mice (*n* = 25), New Zealand rabbits (*n* = 11), and humans (*n* = 48). (B) Intertype specificity. Neutralizing potency of rat sera (*n* = 5 per group) against three serotypes of poliovirus after immunization with a monovalent bulk of Sabin‐IPV 1, 2, and 3 was tested, respectively. (C) Specificity for poliovirus among other viruses. To validate specificity of tricolor PBNA, CV‐A6 (mouse, *n* = 1), CV‐A10 (mouse, *n* = 1), CV‐A16 (mouse, *n* = 1), EV71 (mouse, *n* = 1) enterovirus antibody‐positive sera (mouse, *n* = 1), a SARS‐CoV‐2 antibody‐positive serum (guinea pig, *n* = 1), and H3N2 antibody‐positive serum (guinea pig, *n* = 1) were tested against all three poliovirus (PV) serotype pseudoviruses by PBNA. A poliovirus‐positive rabbit serum (*n* = 1) was used as the positive control. Statistical analysis was performed using one‐way ANOVA followed by Tukey's multiple comparisons test. Significance levels are indicated with asterisks: *p < 0.05, **p < 0.01, ***p < 0.005, ****p < 0.001. (D) Reproducibility with Sabin strains. To validate the reproducibility of tricolor PBNA, serum from a rabbit (*n* = 1) immunized with a trivalent Sabin‐IPV blend was tested using the established fluorescent PBNA. The assay was performed independently three times, each with six replicates. The average intra‐assay and interassay coefficients of variation (CV) were calculated. (E) Reproducibility with WPV and VDPV strains. The experimental procedure was identical to (D) described above, with the exception that WPV1, VDPV2, and WPV3 pseudoviruses were used.

To validate the cut‐off value for the PBNA, we calculated the limit of detection of the PBNA using the Sabin1‐E2, Sabin2‐eGFP, and Sabin3‐RFP pseudoviruses in combination with 104 negative sera (including 20 rat negative sera, 25 mouse negative sera, 11 New Zealand rabbit negative sera, and 48 human sera). The serum was initially diluted threefold, followed by threefold serial dilution, and the remaining steps followed the PBNA. The cut‐off value was defined as the mean plus 1.96 standard deviations (mean + 1.96 SD) [32]. The limit of detection (LOD) of Sabin 1, 2, and 3 poliovirus NAbs was 13.57, 9.18, and 8.18 in rats, 12.41, 6.79, and 4.10 in mice, 12.43, 6.06, and 4.05 in rabbits, and 28.22, 25.08, and 13.04 in humans, respectively. When all 104 negative sera were analyzed together, the LODs were 22.56 for Sabin 1, 19.05 for Sabin 2, and 10.30 for Sabin 3 (Figure 3A). To maintain high specificity and establish a single, conservative threshold for all three serotypes, a cut‐off value was set at 30.

#### 2.2.5 Validation of the Specificity of the PBNA

To verify the intertype specificity of the method, we tested the neutralization potency of rat sera against the three poliovirus serotypes after immunization with 15 preparations of monovalent bulk of Sabin‐IPV (5 each of types 1, 2, and 3), respectively. As shown in Figure 3B, the serum after immunization with monovalent bulk of Sabin‐IPV (type 1) could only neutralize Sabin1‐E2 pseudovirus, and the serum after immunization with monovalent bulk of Sabin‐IPV (type 2) could only neutralize Sabin2‐eGFP pseudovirus. Similarly, serum after immunization with a monovalent bulk of Sabin‐IPV (type 3) neutralized only Sabin3‐RFP pseudovirus (Figure 3B), suggesting that the method had excellent intertype specificity. To verify the specificity of the method among different viruses, we tested the neutralization potency of CV‐A6, CV‐A10, CV‐A16, and EV71 enterovirus antibody‐positive sera (mouse), SARS‐CoV‐2 antibody‐positive sera (guinea pig), and H3N2 antibody‐positive sera (guinea pig) against the three serotypes of pseudovirus, with poliovirus‐positive sera (rabbit) as the positive control. As shown in Figure 3C, only poliovirus antibody‐positive serum was able to neutralize the three serotypes of poliovirus, while none of the other sera could neutralize the pseudoviruses (Figure 3C), suggesting that the method also had good specificity for poliovirus among different viruses.

#### 2.2.6 Validation of the Reproducibility of the PBNA

To verify the reproducibility of the method, we performed the PBNA on 1 rabbit serum after immunization with the trivalent blend of Sabin‐IPV. The procedure was repeated three times independently with reference to the pseudovirus neutralization, and six replicates were done for each assay to calculate the average coefficient of variation (CV) within and between tests. As shown in Figure 3D, the intratest CVs of Sabin type 1–3 neutralization antibody assays were 12.52%, 12.08%, and 11.18%, respectively. Similarly, the intertest CVs were 13.39%, 14.65%, and 11.87%, respectively (Figure 3D). Additionally, to evaluate the reproducibility of this assay and confirm its applicability to epidemiologically relevant poliovirus strains, we also determined NAbs titers against the WPV1 strain (GenBank accession numbers KU866422, Mahoney), vaccine‐derived polioviruses (VDPVs) type 2 strain (GenBank accession numbers JX275147), and WPV3 strain (GenBank accession numbers KP247597, Saukett). The procedure was identical to the one described above. As shown in Figure 3E, the intra‐assay CVs for the three strains were 7.90%, 7.76%, and 5.13%, respectively, while the interassay CVs were 10.11%, 10.10% and 6.15%, respectively. To assess interbatch consistency of pseudovirus performance, we tested the same Sabin‐IPV‐immunized rabbit serum using three batches of Sabin type 1, 2, and 3 pseudoviruses. The calculated interbatch CVs were 5.01%, 9.95%, and 7.98% for Sabin1, 2, and 3, demonstrating high consistency among pseudovirus batches (Figure S1). Based on these results, the method exhibited good reproducibility.

### 2.3 Correlation of the PBNA with the Gold‐Standard cNT

**FIGURE 4:**
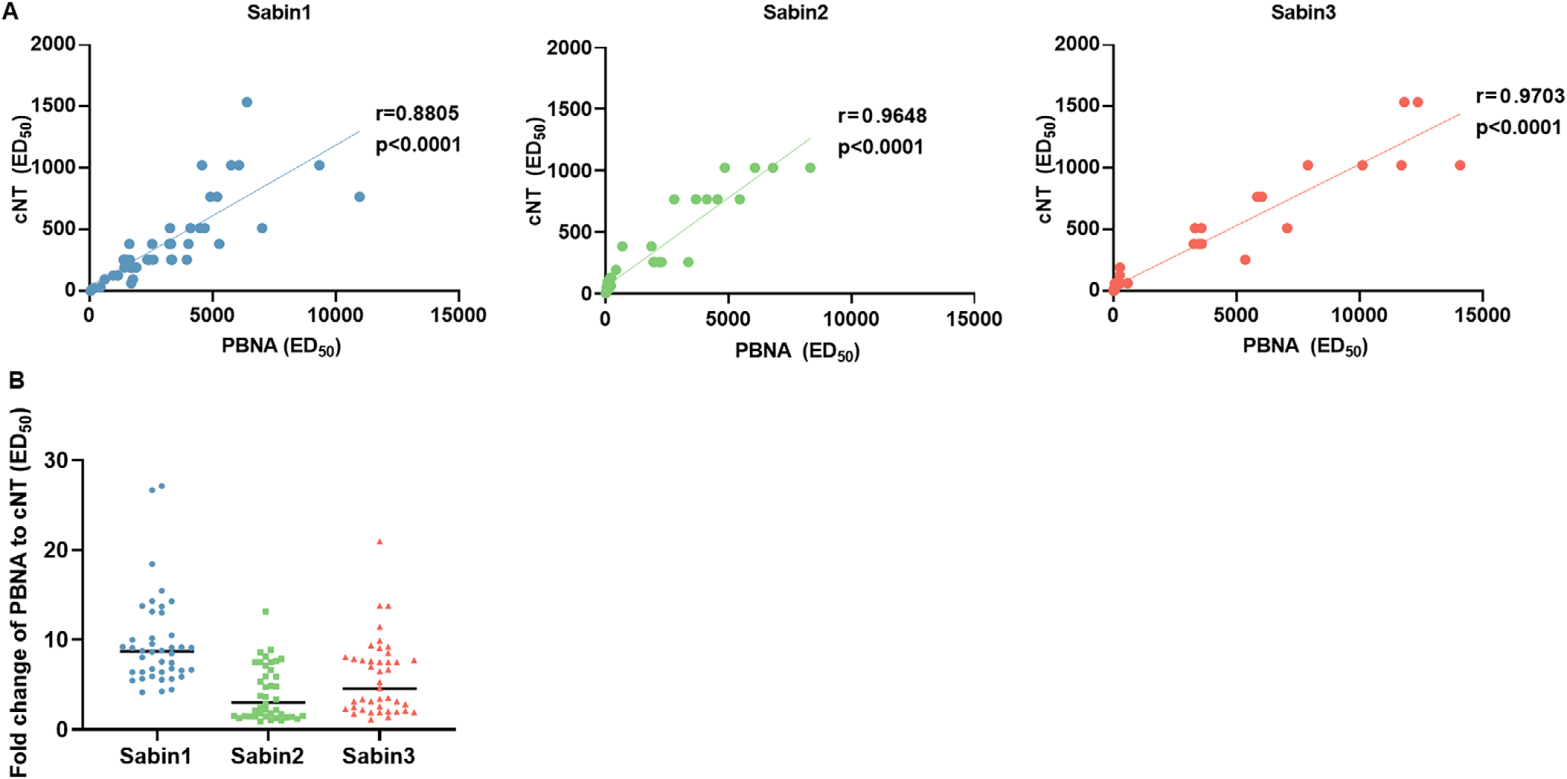
Analysis of the correlation between the PBNA and cNT using animal sera. (A) Correlation analysis between PBNA and cNT. Neutralizing antibodies against Sabin 1, 2, and 3 were detected in 42 trivalent blends of Sabin‐IPV immunized sera (rabbit, *n* = 15; rat, *n* = 27) using the PBNA and cNT, respectively, after which the ED_50_ values of Sabin 1, 2, and 3 measured by the above two assays were subjected to Spearman correlation analysis. Values below the detection limit were assigned a value equal to half of that limit for statistical analysis. The trend line is represented by a solid line in the graph, r indicates the correlation between the two assays, and *p* < 0.0001 indicates a significant difference in the results. (B) Fold‐difference of ED_50_ values between PBNA and CNT. The Y‐axis indicates the ratio of PBNA versus cNT ED_50_ values for Sabin 1, 2, and 3. The horizontal line indicates the geometric mean of the multiplicity of ED_50_ values for different serotypes.

**FIGURE 5:**
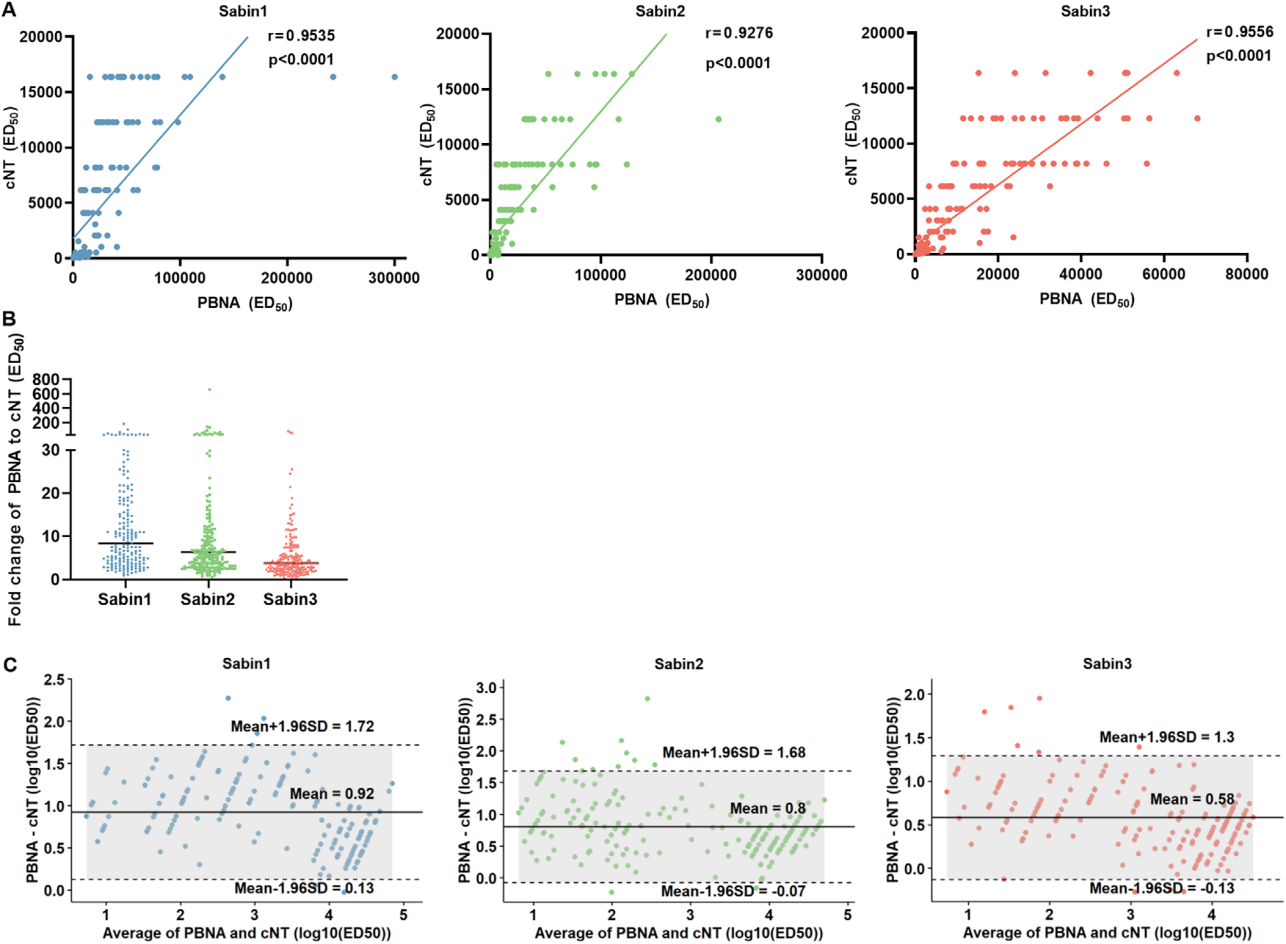
Analysis of the correlation between PBNA and cNT using human sera. (A) Correlation analysis of the PBNA and cNT. The neutralizing antibodies against Sabin 1, 2, and 3 were detected in 211 postimmunization human sera (clinical booster‐immunized sera, *n* = 111; healthy donor sera from Shandong Province, *n* = 100) using PBNA and cNT. The ED_50_ values for Sabin 1, 2, and 3 measured by the above two assays were subjected to Spearman correlation analysis. Sera with cNT ED_50_ values above the upper detection limit were excluded from the correlation analysis. Values below the detection limit were assigned a value equal to half of that limit for statistical analysis. The trend line is represented by a solid line in the graph, r indicates the correlation between the two assays, and *p* < 0.0001 indicates a significant difference in the results. (B) Fold difference of ED_50_ values between the PBNA and cNT. The Y‐axis indicates the ratio of pseudovirus and cNT ED_50_ values of Sabin 1, 2, and 3. The horizontal line indicates the geometric mean of the multiplicity of ED_50_ for different serotypes. (C) Bland–Altman analysis of assay consistency. A Bland‐Altman plot was used to compare the log10‐transformed ED_50_ values obtained from the PBNA and cNT. The solid line represents the mean difference between the two methods, while the dashed lines indicate the limits of agreement.

To further evaluate the reliability of the tricolor fluorescence‐based poliovirus PBNA, we compared its calculated NAbs titers against those obtained from the gold‐standard cNT for poliovirus serotypes 1, 2, and 3. A total of 253 serum samples were analyzed, including 42 trivalent Sabin‐IPV postimmunization animal sera (15 rabbit‐derived and 27 mouse‐derived samples) as well as 211 human sera (111 from Phase III clinical trials following Sabin‐IPV booster immunization and 100 from healthy individuals in Shandong Province). Sera with cNT ED_50_ values above the upper detection limit (cNT ED_50_ values >16384) were excluded from the correlation analysis. This left 180, 201, and 208 human sera for both assays for poliovirus types 1–3, respectively. Values below the detection low limit were assigned a value equal to half of that limit for statistical analysis (PBNA ED_50_ values <30 were imputed as 15; cNT ED_50_ values <4 were recorded as 2, and values <8 as 4). As shown in Figure 4A, strong correlations among animal sera were observed between the PBNA and cNT for Sabin types 1, 2, and 3, with Spearman correlation coefficients of 0.8805, 0.9648, and 0.9703 (*p *< 0.0001), respectively (Figure 4A). However, the ED_50_ values of PBNA were higher than those from the cNT, by 8.72‐, 3.02‐, and 4.56‐fold for Sabin types 1, 2, and 3, respectively (Figure 4B). Nevertheless, a strong correlation was observed in human sera, with correlation coefficients of 0.9535, 0.9275, and 0.9556 (*p *< 0.0001) for serotypes 1, 2, and 3, respectively (Figure 5A). The corresponding ED_50_ values obtained using the PBNA were 8.38, 6.37, and 3.82 times higher than those derived from the cNT (Figure 5B). We then evaluated the correlation between tricolor PBNA and single‐color PBNA using 15 rabbit serum samples (Figure S2). Both assays demonstrated strong Spearman correlations for Sabin 1, 2, and 3 of 0.9357, 0.9250, and 0.8071 (all *p *< 0.05). Furthermore, the comparison revealed consistent mean‐fold changes of 0.68, 0.82, and 0.73 for types 1, 2, and 3, respectively.

To further assess the consistency between PBNA and cNT, we employed a combined approach of bivariate analysis (negative, positive) and continuous variable analysis (ED_50_ values). Based on ED_50_ cut‐off values for positive/negative determination, results showed concordance rates of 98.8%, 92.5%, and 93.3% for PV1, PV2, and PV3, respectively, all exceeding 90%. Subsequently, we performed Bland–Altman analysis using log10‐transformed ED_50_ values, including the difference and the mean between PBNA and cNT, to confirm the consistency (Figure 5C). The limits of agreement (mean ± 1.96 SD) for all three serotypes are as follows: PV1 [0.13, 1.72] (mean, 0.93; SD, 0.41), PV2 [−0.07, 1.68] (mean, 0.81; SD, 0.45), and PV3 [−0.13, 1.3] (mean, 0.59; SD, 0.37). Except for a few outliers, the vast majority of samples fell within these limits, further supporting the consistency between the two assays.

### 2.4 Factors Influencing Poliovirus NAbs Titers in Healthy Individuals from Shandong Province

To evaluate the impact of demographic factors on the levels of NAbs against the three serotypes of poliovirus, we employed GEE to assess the associations of age, sex, and residence with antibody titers. The results are shown in Table 1. For PV1, individuals aged 10–19 years (*β* = –384.70, *p* < 0.001) and >19 years (*β* = –411.83, *p* < 0.001) exhibited significantly lower NAbs levels in the cNT compared with those <10 years. A similar but more pronounced trend was observed in the PBNA, where the 10–19 (*β* = –7250, *p* < 0.001) and >19 (*β* = –7330, *p* < 0.001) age groups showed markedly reduced neutralizing titers. For PV2, the results of cNT showed that both the 10–19 (*β* = –101.63, *p* < 0.001) and >19 (*β* = –97.96, *p* < 0.001) age groups had significantly lower antibody levels than the <10 age group. In the PBNA, the 10–19 age group had significantly reduced titers (*β* = –879, *p* = 0.012), while the >19 groups also showed a decreasing trend (*β* = –519, *p* = 0.385), albeit without statistical significance. For PV3, the results mirrored those of PV1, with significantly lower antibody levels observed in the 10–19 (*β* = –353.6, *p* < 0.001) and >19 (*β* = –370.4, *p* < 0.001) groups compared with the <10 group. The PBNA again revealed a more pronounced decline in neutralizing titers in the 10–19 (*β* = –2446.3, *p* < 0.001) and >19 (*β* = –2610.2, *p* < 0.001) age groups. For PV 1, 2, and 3, no significant differences were found based on sex or residence (all *p* > 0.05). In summary, age emerged as the primary determinant of NAbs levels against all three poliovirus serotypes.

**TABLE 1:** GEE analysis of the factors related to the ED_50_ levels observed in the cNT and PBNA with sera from healthy volunteers from Shandong Province.

		cNT		PBNA	
Serotype	Factors	β (95% CI)	*p‐*value	β (95% CI)	*p‐*value
PV1	Age				
	<10	1.00 (reference)		1.00 (reference)	
	10–19	−384.70 (−522.08, −247.32)	<0.001	−7250 (−9472, −5028)	<0.001
	>19	−411.83 (−552.20, −271.46)	<0.001	−7330 (−9970, −4690)	<0.001
	Sex				
	Male	1.00 (reference)		1.00 (reference)	
	Female	−34.90 (−159.56, 89.76)	0.582	−656 (−2754, 1442)	0.540
	Location				
	Urban	1.00 (reference)		1.00 (reference)	
	Rural	81.95 (−61.62, 225.52)	0.265	3168 (521, 5815)	0.019
PV2	Age				
	<10	1.00 (reference)		1.00 (reference)	
	10–19	−101.63 (−141.96, −61.30)	<0.001	−879 (−1570, −188)	0.012
	>19	−97.96 (−149.52, −46.40)	<0.001	−519 (−1690, 652)	0.385
	Sex				
	Male	1.00 (reference)		1.00 (reference)	
	Female	3.81 (−37.32, 44.94)	0.856	−424 (−1149, 301)	0.246
	Location				
	Urban	1.00 (reference)		1.00 (reference)	
	Rural	54.89 (−1.92, 111.70)	0.058	908 (−134, 1950)	0.088
PV3	Age				
	<10	Reference		Reference	
	10–19	−353.6 (−488.9, −218.3)	<0.001	−2446.3 (−3504.3, −1388.3),	<0.001
	>19	−370.4 (−513.8, −227.0)	<0.001	−2610.2 (−3712.6, −1507.8)	<0.001
	Sex				
	Male	Reference		Reference	
	Female	−48.7 (−185.6, 88.2)	0.486	−50.1 (−1197.2, 1097.0)	0.932
	Location				
	Urban	Reference		Reference	
	Rural	52.3 (−117.9, 222.5)	0.545	489.3 (−847.8, 1826.4)	0.473

Abbreviations: CI: confidence interval; cNT: the conventional neutralization test; PBNA: the pseudovirus‐based neutralization test; PV: poliovirus.

## 3 Discussion

The detection of poliovirus NAbs is crucial for monitoring population immunity and evaluating vaccine efficacy. At present, the detection methods of poliovirus NAbs mainly include cNT, ELISA, and PBNA based on the luciferase reporter gene. The cNT is the gold standard for the detection of poliovirus NAbs [12], but with the progressive sequestration of polio, it is becoming increasingly difficult to use infectious authentic viruses, requiring dedicated biosafety facilities. Moreover, because of its cumbersome operation and long detection period, it is unsuitable for large‐scale population‐based immunity monitoring. In response to these challenges, alternatives to the cNT assay have been developed, such as ELISA and PBNA, which do not require infectious viruses, offer shorter assay times, and have lower biosafety requirements. ELISA is based on principles of nonfunctional binding, blockade, or binding inhibition and is commonly used to evaluate poliovirus antibodies in vaccine research and epidemiological studies [13]. However, ELISA primarily measures the binding of antibodies to specific antigens, which does not accurately reflect their neutralizing effect. Additionally, the assay is limited by factors such as low sensitivity, narrow linear range, dose dependence, and weak correlation with the results of authentic virus neutralization assays [13, 14, 15]. By contrast, the PBNA is an attractive alternative to the traditional neutralization assay. Compared with cNT, PBNA has several notable advantages, as it enables the synthesis of a pseudovirus with any known sequence or point mutation without the need for infectious authentic viruses. Moreover, the PBNA significantly shortens the assay time to approximately 12 h. As it incorporates a reporter gene, it also allows for objective quantification, and it requires lower biosafety levels. However, the existing chemiluminescence‐based PBNA protocols can only detect one poliovirus serotype at a time, resulting in low throughput. Additionally, it requires the addition of costly luciferase substrates, making it expensive.

To address the high cost and complexity of existing chemiluminescent PBNA, which rely on expensive substrates, we developed the fluorescent pseudovirus system. A prerequisite for this was establishing a high‐titer production protocol. In this study, fluorescent poliovirus pseudovirus was prepared by cotransfection of HEK 293T cells with the P1 plasmid, a plasmid replicon carrying a fluorescent reporter gene, and a T7 RNA polymerase plasmid. Since it has been shown that the Kozak consensus sequence can improve RNA stability and translation initiation level [33], we inserted the Kozak sequence in front of P1 in the pcDNA3.4 vector to enhance the expression of the P1 protein. Similarly, Liang et al. [32] successfully constructed a high‐titer fluorescent SARS‐CoV‐2 pseudovirus system by introducing Kozak sequences to optimize translation initiation and enhance target protein expression. To enhance the pseudovirus titer, we optimized the preparation conditions of the pseudovirus and determined that the optimal transfection ratio of the above three plasmids was 3:3:1, the most sensitive cell line was HEK 293T, and the P1 plasmid was modified with a Kozak‐P1 sequence. Notably, this resulted in a significant enhancement of the pseudovirus titer by more than 29‐fold. Importantly, the high tolerance of the pseudoviruses to repeated freeze‐thaw cycles (up to six cycles at −80°C) confirms the stability of pseudoviruses. In conclusion, we successfully prepared stable, high‐titer pseudoviruses of three poliovirus serotypes carrying different fluorescent protein genes (E2, eGFP, RFP). The fluorescent pseudovirus system offers clear advantages. It significantly reduces costs by being substrate‐free, which also simplifies the workflow and minimizes variability. Its stable signal also allows for flexible measurement timing, unlike the transient signals from chemiluminescent reactions that demand precise timing. Most importantly, the availability of diverse fluorescent proteins with distinct spectral properties enables multiplexing [34]. This capability was key to developing our assay for the concurrent measurement of NAbs titers against all three poliovirus serotypes. This capability is particularly advantageous for poliovirus, which comprises three distinct serotypes (PV1, PV2, and PV3) that do not confer cross‐immunity. We leveraged this to establish a high‐throughput assay for the concurrent measurement of NAbs titers against all three serotypes.

The challenge for tri‐color PBNA was managing potential signal interference. We encountered two major issues that could lead to counting ambiguity: spectral overlap between fluorescent reporter genes and the possibility of biological cross‐reactions or co‐infections. As detailed in the supplementary data (Table S1), we evaluated nine distinct fluoroproteins to identify a triplet with minimal spectral overlap and the highest positive cell numbers. We ultimately selected eGFP, RFP, and E2, which are optimally excited and detected in three independent, well‐separated filter channels (Figure 1B). This step was crucial for minimizing optical counting errors. This multiplex approach using distinct fluoroproteins has also been successfully applied to other viruses like HPV and Dengue. For example, Nie et al. [35] utilized three fluorescent proteins for multiplex neutralization assays against different HPV serotypes, while Matthias et al. [36] established a similar system for all four dengue virus serotypes. At the biological level, we validated specificity using monovalent sera. As shown in Figure 3B, serum targeting a specific serotype neutralized only the corresponding pseudovirus, with no cross‐neutralization observed. The combination of strict spectral separation and validated biological specificity effectively avoided counting ambiguity, confirming the reliability of our multiplex detection method.

Then, we established a high‐throughput PBNA that can simultaneously detect all three poliovirus serotypes in a single assay, reducing sample consumption and improving efficiency. Furthermore, to establish the assay's high‐throughput capacity, we optimized the automated workflow by integrating the BioTek Cytation 5 reader and Stacker 4 robotic arm. The optimized system achieved a consistent 10 min cycle time per 96‐well plate, covering automated loading, tri‐channel fluorescence imaging, and data export. This allows a single instrument to process 48 plates within a standard 8 h daily operation. For our tri‐plex assay format (8 samples/plate), this capacity equates to a daily throughput of 384 serum samples, generating 1152 data points. Notably, these calculations are based on a single system; this capacity can be readily and significantly scaled by operating multiple systems in parallel. Although its high‐throughput automation is a major advantage when processing large‐scale samples, it must be emphasized that automation is not a prerequisite. The entire detection method was initially developed and validated using standard manual experimental procedures, including single‐channel or multichannel pipetting and manual monitoring. This confirms the method's seamless adaptability to standard laboratories with lower equipment density, facilitating its broad adoption in both small‐scale studies and large‐scale screenings.

We established a high‐throughput method for the combined detection of NAbs against all three poliovirus serotypes. The optimal parameters of the method were found to encompass a detection time of 12 h, seeding with HEK 293T cells at a density of 4 × 10^4^ cells/well, and a pseudovirus dose of 1500 TCID_50_/mL. To validate the cut‐off value, negative sera from rats, mice, rabbits, and humans were incorporated to ensure high specificity, and the final cut‐off value was set at 30. When intertype specificity was verified, the results showed that sera elicited by a monovalent bulk of Sabin‐IPV (type 1) could only neutralize Sabin1‐E2 pseudovirus, with corresponding results for Sabin2 and Sabin3, respectively, demonstrating the excellent intertype specificity of the method. Similarly, Jiang et al. [31] confirmed the noncrossover between the WPV strains Mahoney, MEF, and Saukett with sera from mice immunized with a monovalent vaccine. When the specificity between different viruses was verified, the results showed that only poliovirus antibody‐positive sera were able to neutralize the pseudoviruses corresponding to the three poliovirus serotypes, while all other sera were nonreactive, which demonstrated the good specificity of the method for poliovirus among different enteroviruses. The assay is highly reproducible, a finding confirmed not only with Sabin vaccine strains (all CVs <15%) but also, importantly, with WPV and VDPV strains (Figure 3E). The excellent interbatch reproducibility of our pseudovirus production, with CVs under 10% for all three serotypes (Figure S1), confirms that our method consistently yields reliable virus preparations and ensures the stability of neutralization results across different experiments. High interassay consistency and freeze‐thaw stability are critical for ensuring the reliability and reproducibility of neutralization results, highlighting the robustness and practical value of this assay system. The method is a reliable tool for large‐scale surveillance and immunity monitoring in the post‐polio era.

The correlation between the PBNA established in this study and the results of the cNT was greater than 0.88 for all three types. However, the ED_50_ values of PBNA were higher than those of cNT, showing that the pseudotyped poliovirus was more sensitive than the authentic virus. The inconsistency in the geometric mean of the values for the three poliovirus serotypes may be due to the characteristics of the virus itself. Additionally, our tricolor PBAN also demonstrated excellent correlation with the single‐color PBNA (*r* > 0.80, *p* < 0.05). Consistency analysis demonstrated that all three serotypes achieved high agreement rates (>92%), suggesting a strong concordance between the PBNA and cNT. Bland–Altman analysis further confirmed the high concordance between the two detection methods, with results showing that nearly all paired measurements fell within the limits of agreement, except for a few outliers. Nevertheless, all the validation results suggest that this method can be used as an alternative to the cNT. In this study, we investigated the factors influencing poliovirus NAbs titers among healthy individuals in Shandong Province. Our results identified age as the primary determinant, with GEE analysis revealing a significant decline in antibody titers with increasing age, indicating evident immune waning or immunity gaps among adolescents and adults. Similarly, data from a cross‐sectional study involving 2611 individuals during a WPV outbreak in Xinjiang Uygur Autonomous Region of China revealed notably lower seropositivity rates and geometric mean titers among adults aged 15–39 years [37]. Supporting this observation, surveillance in 2011 indicated that 52.38% of WPV cases imported from Pakistan into Xinjiang occurred in individuals aged 15 years and older [37]. These findings underscore the importance of closely monitoring poliovirus NAbs levels not only in children but also in adolescents and adults. Such age‐related differences in immunity present a notable public health risk, especially in the context of incomplete global eradication of poliovirus. Given China's proximity to countries where WPV remains endemic, such as Afghanistan and Pakistan, the risk of imported outbreaks persists. In addition, challenges posed by VDPVs further complicate the situation. In summary, to effectively prevent potential outbreaks caused by VDPVs or imported WPV, it is necessary to close the immunity gap among adolescents and adults. In addition to maintaining high routine immunization coverage in children, regular supplementary immunization efforts should be prioritized for adolescents and adults, particularly in high‐risk regions. In contrast, age, sex, and residence were not significantly associated with antibody levels, suggesting relatively uniform vaccine coverage across demographic groups. The strong concordance between PBNA and cNT results supports the former as a safe and reliable alternative for poliovirus antibody surveillance.

However, we acknowledge certain limitations of this study. Although we validated the method using diverse serum samples, including those from animals and humans, the sample size was moderate and lacked broad geographical diversity. Furthermore, this validation was conducted in a single laboratory. Therefore, future large‐scale, multicenter validation studies are essential. Such studies should incorporate diverse human serum cohorts from multiple regions and across a broad range of antibody titers to confirm the method's broad utility, robustness, and interlaboratory consistency.

In summary, we established a multiplex neutralization assay for the simultaneous detection of type 1, 2, and 3 poliovirus NAbs and applied it to a cross‐sectional analysis of serum samples from healthy individuals. The method has many advantages, such as high detection throughput, decreased sample usage, low cost, convenient operation, and automated detection. The method can provide a technical tool for detecting NAbs against poliovirus in large‐scale populations, as well as the development and evaluation of novel vaccines to support the poliovirus eradication program.

## 4 Materials and Methods

### 4.1 Cells, Sera, and Plasmids

RD, HEK 293T, Hep2, Vero, and Huh‐7 cells were cultured in Dulbecco's modified Eagle's medium (GIBCO, Grand Island, NY, USA) containing 100 U/mL of penicillin‐streptomycin solution (GIBCO), 20 mM HEPES (N‐2‐hydroxyethylpiperazine‐N‐2‐ethane sulfonic acid, GIBCO), and 10% fetal bovine serum (FBS; PAN‐Biotech, Adenbach, Germany), at 37°C in a 5% CO_2_ cell culture incubator and passaged once every 2–3 days.

A total of 27 trivalent positive sera were collected from Wistar rats after 21 days of immunization with the formulated trivalent bulk of Sabin‐strain inactivated poliovirus vaccine (Sabin‐IPV). An additional 15 univalent positive sera were obtained from Wistar rats, with five animals each immunized for 21 days with a monovalent bulk of Sabin‐strain inactivated poliovirus vaccine (Sabin‐IPV). Fifteen trivalent positive sera from rabbits were collected after 42 days of immunization with the trivalent blend of Sabin‐IPV mixed 1:1 with Freund's complete adjuvant. Single positive serum samples from mice immunized with CV‐A6, CV‐A10, CV‐A16, and EV71, as well as one SARS‐CoV‐2‐positive and one H3N2‐positive guinea pig serum, were also included. Negative controls consisted of 20 rats, 25 mice, and 11 New Zealand white rabbit sera, 48 human sera, totaling 104 samples. Human serum samples were obtained from individuals who had received booster immunization with Sabin‐IPV and from healthy volunteers in Shandong Province, China.

The eukaryotic expression vectors used in this study were derived from Invitrogen's pcDNA3.4 vector and Promega's pGEM‐T Easy Vector. The poliovirus P1 gene was codon optimized, and the Kozak sequence (GCCACC) was added in front of the start codon ATG in the pcDNA3.4 vector, resulting in the capsid protein expression plasmid (Figure 1A). The capsid protein expression plasmid encodes the specific capsid proteins individually from a specific poliovirus strain. The 5'UTR, different fluorescent reporter genes (eGFP, ZsGreen, YPet, RFP, mOrange, dTomato, mCherry, E2‐crimson (referred to as E2), and mKate2), the P2 gene, the P3 gene, and the 3'UTR were inserted behind the T7 promoter of the pGEM‐T Easy Vector, and a 2Apro site was introduced behind the fluorescent reporter gene, with Poly(A) added to the end of the 3'UTR, resulting in the plasmid replication (Figure 1A). The T7 RNA polymerase gene was inserted into the pcDNA3.4 vector, resulting in the T7 RNA polymerase plasmid (Figure 1A). To ensure high sequence fidelity and reproducibility, all plasmids in the study were produced using commercial gene synthesis using poliovirus sequences. The sequences employed were sourced from the NCBI database, with GenBank accession numbers provided in the text. No specific primers targeting poliovirus sequences were utilized.

### 4.2 Pseudovirus Packaging

The P1 plasmid, replicon plasmid, and T7 RNA polymerase plasmid were used to co‐transfect HEK 293T cells at 60∼70% confluence according to the instructions of the Lipofectamine 3000 transfection reagent (Invitrogen, L3000015). The medium was changed (2% FBS) after 4∼6 h of transfection. After 48 h of infection, the cells and culture supernatant were frozen and thawed twice. The cell debris was removed by centrifugation at 4000*g* for 30 min, after which the supernatant was filtered through a 0.22 µm pore‐size membrane and stored at −80°C until further use. A schematic diagram of the workflow for fluorescent pseudovirus production is shown in Figure 1A.

### 4.3 Pseudovirus Titration

In 60 wells (B2‐G11) of a 96‐well plate, 100 µL of culture medium was added. In 6 wells (B2‐G2), 50 µL of prepared polio pseudovirus was added. Then, starting from well B2‐G2, a threefold serial dilution was performed to wells B3–G3 and each subsequent well in sequence, whereby 50 µL of mixed liquid was removed from the final wells B10‐G10 and discarded. Then, 100 µL of HEK 293T cell suspension (4 × 10^4^ cells/well) was added into the middle 60 wells of the 96‐well plate, while the outermost wells were filled with 250 µL of sterile water. The 96‐well plate was incubated in an atmosphere comprising 5% CO_2_ at 37°C for 12 h, after which the number of fluorescent spots was quantified (Cytation5, BioTek). When determining the titers of three types of pseudoviruses, pseudovirus stocks were prepared in threefold serial dilutions, and each dilution was inoculated into six replicate wells. After a 12 h incubation at 37°C, the number of fluorescent spots was quantified using BioTek, and titers were calculated by the Reed–Muench method. The final titers were expressed as 50% tissue culture infectious dose per milliliter (TCID_50_/mL).

### 4.4 Pseudovirus‐Based Neutralization Assay

After inactivating the serum samples and serial dilution, 100 µL of the diluted samples and 50 µL of pseudovirus were added to each well of a 96‐well plate (the amount of pseudovirus added was 1500 TCID_50_/mL). After incubation at 37°C for 1 h, 4 × 10^4^ HEK 293T cells were added to each well. Then, the 96‐well plate was incubated in a humidified atmosphere comprising 5% CO_2_ at 37°C for 12 h. After a 12 h incubation, 96‐well plates were imaged using a BioTek Cytation 5 with a Stacker 4 robotic arm for automated handling. Images were acquired using three filter sets: GFP (Ex 469 nm/Em 525 nm), RFP (Ex 531 nm/Em 593 nm), and E2 (Ex 628 nm/Em 685 nm), individually. The number of fluorescent pseudovirus‐infected cells per well was quantified using Gen5 software with optimized “Primary Mask and Count” settings for each channel: GFP (threshold 7000, object size 10–150 µm), RFP (threshold 7000, size 5–50 µm), and E2 (threshold 8800, size 5–50 µm). These counts were then used to calculate neutralization percentages. The 50% Effective Dose (ED_50_) was calculated using the Reed‐Muench method or nonlinear regression. The ED_50_ represents the dilution of the sample at 50% inhibition.

### 4.5 The Authentic Virus Neutralization Assay

Sera were inactivated at 56°C for 30 min to detect NAbs against the three serotypes of poliovirus. Specifically, a twofold serial dilution of serum was performed in 96‐well culture plates, 50 µL per well, and then 50 µL of l00 CCID_50_ virion suspension of the corresponding serotype was added to each well, and neutralized at 37°C for 3 h. After discarding the culture supernatant, Hep2 cells that had grown into a healthy monolayer were digested for 2 min minutes and the digestive solution was discarded, after which 5 mL of culture medium were added to disperse the cells for counting, Then, the concentration of the cells with the culture medium to contain 5×10^4^∼10×10^4^ cells per milliliter, and 100 µL of the resulting cell suspension were added to each well of the neutralized 96‐well cell culture plate, and incubated at 35.5°C in a humidified atmosphere comprising 5% CO_2_ for 7 days. Cytopathic lesions were observed and recorded under the microscope. The highest dilution of serum in which 50% of the cell wells did not produce lesions was taken as the endpoint, and the reciprocal of the dilution was taken as the NAbs potency.

### 4.6 Data Analysis

Data were analyzed using GraphPad Prism version 8.0 (GraphPad Software Inc., San Diego, CA, USA) and R software version 4.4.2 (R Foundation for Statistical Computing, Vienna, Austria). Schematic diagrams illustrating the experimental design were created with BioRender (biorender.com). Experimental data are presented as the means ± standard error of the mean (SEM), based on three independent replicates. For datasets with varying sample sizes, results were expressed as geometric means. For pairwise comparisons, Student's *t*‐test was employed. One‐ or two‐way analysis of variance (ANOVA) was used for comparisons among multiple groups. When statistically significant differences were observed, the multiple comparisons test was conducted to identify specific group differences. The Spearman correlation coefficient and generalized estimating equations (GEE) were employed to assess the correlation between neutralization results obtained from PBNA and cNT. In addition, GEE was applied to evaluate the associations between demographic factors (including age, sex, and region) and NAbs levels, assuming an independent working correlation structure and Gaussian distribution. All models were adjusted for relevant covariates. Significance levels are indicated with asterisks: *p <0.05, **p <0.01, ***p <0.005, ****p <0.001.

## Author Contributions

Youchun Wang conceived, designed, and supervised the experiments. Li Zhang, Lei Ma, Li Yi, and Guoyang Liao provided the clinical and animal samples. Meiyan Liu, Yadong Li, Zexin Tao, Lan Huang, Xi Wu, Yong Zhang, Shuangli Zhu, Qiang Sun, Tianjiao Ji, Dongyan Wang, Ziteng Liang, Shuo Liu, Meina Cai, Yimeng An, and Jierui Li performed the experiments. Meiyan Liu, Yuanling Yu, Weijin Huang, and Guoyang Liao analyzed the experimental data. Meiyan Liu and Yuanling Yu wrote the manuscript. Youchun Wang, Li Zhang, Lei Ma, Li Yi, and Weijin Huang revised the manuscript. All authors have read and approved the final manuscript.

## Funding

This study was financially supported by the CAMS Innovation Fund for Medical Sciences (project nos. 2022‐I2M‐3‐001), the Changping laboratory, the Public Health Talent Development Support Program of the National Disease Control and Prevention Administration, and the Taishan Scholar Project of Shandong Province (tstp20221164).

## Conflicts of Interest

The authors declare no conflicts of interest.

## Ethics Statement

The study protocols for human serum samples were approved by the Ethics Committee of the Guangxi CDC (ethics approval number: IRB00001594) and the Ethics Committee of the Shandong CDC (ethics approval number: SDJK2025‐007‐01). All participants provided written informed consent, and all experiments involving human sera were performed according to the Code of Ethics of the World Medical Association (Declaration of Helsinki). The experiments on animals were conducted according to the guidelines of the Institutional Animal Care and Use Committee at the Institute of Medical Biology, Chinese Academy of Medical Sciences (CAMS). The animal experiments were approved by its ethics committee (ethics number: DWSP202401004).

## Supporting information




**Supporting Table 1**: Titers of poliovirus pseudoviruses containing different fluorescent protein genes under different excitation wavelengths.
**Supporting Figure 1**: Interbatch consistency of pseudovirus performance. To evaluate batch consistency of pseudovirus, we used three independently prepared batches of Sabin types 1, 2, and 3 pseudovirus to detect the neutralizing titer (ED_50_) of the same Sabin‐IPV immunized rabbit serum (*n* = 1) via the PBNA. The X‐axis labels 1, 2, and 3 represent the three independent pseudovirus batches with six replicate wells per assay. The calculated interbatch CVs were 5.01%, 9.95%, and 7.98% for types 1, 2, and 3, respectively, demonstrating high consistency among different pseudovirus batches in the assay application.
**Supporting Figure 2**: Analysis of the consistency between tricolor PBNA and single‐color PBNA. (A) Correlation analysis. Neutralizing titers (ED_50_) against poliovirus types 1, 2, and 3 from rabbit sera (*n* = 15) were measured using both the tricolor PBNA and single‐color PBNA. The ED_50_ values of two assays were then compared using Spearman correlation analysis. The trend line is represented by a solid line in the graph, r indicates the correlation between the two assays, and *p* < 0.05 indicates a significant difference in the results. (B) Fold‐difference between tricolor PBNA and single‐color PBNA. The Y‐axis shows the fold change, calculated as the ratio of the ED_50_ from the tricolor PBNA to that from the single‐color PBNA. The horizontal line indicates the geometric mean of the ED_50_ for different serotypes.

## Data Availability

The data supporting the results of this study are available from the corresponding author upon reasonable request.
