# Supplementary material for: A Pseudovirus‐Based Method for the Simultaneous Quantitation of Neutralization Antibodies Against All Three Poliovirus Serotypes
Source: MedComm (2020). 2025 Dec 17;6(12):e70551. doi: 10.1002/mco2.70551 (PMC12710070; doi:10.1002/mco2.70551)
Supplement: Supplementary file 1 — Supporting Table 1: Titers of poliovirus pseudoviruses containing different fluorescent protein genes under different excitation wavelengths. Supporting Figure 1: Interbatch consistency of pseudovirus performance. To evaluate batch consistency of pseudovirus, we used three independently prepared batches of Sabin types 1, 2, and 3 pseudovirus to detect the neutralizing titer (ED50) of the same Sabin‐IPV immunized rabbit serum (n = 1) via the PBNA. The X‐axis labels 1, 2, and 3 represent the three independent pseudovirus batches with six replicate wells per assay. The calculated interbatch CVs were 5.01%, 9.95%, and 7.98% for types 1, 2, and 3, respectively, demonstrating high consistency among different pseudovirus batches in the assay application. Supporting Figure 2: Analysis of the consistency between tricolor PBNA and single‐color PBNA. (A) Correlation analysis. Neutralizing titers (ED50) against poliovirus types 1, 2, and 3 from rabbit sera (n = 15) were measured using both the tricolor PBNA and single‐color PBNA. The ED50 values of two assays were then compared using Spearman correlation analysis. The trend line is represented by a solid line in the graph, r indicates the correlation between the two assays, and p < 0.05 indicates a significant difference in the results. (B) Fold‐difference between tricolor PBNA and single‐color PBNA. The Y‐axis shows the fold change, calculated as the ratio of the ED50 from the tricolor PBNA to that from the single‐color PBNA. The horizontal line indicates the geometric mean of the ED50 for different serotypes. [file MCO2-6-e70551-s001.docx]

**A pseudovirus-based method for the simultaneous quantitation of neutralization antibodies against all three poliovirus serotypes**

**Meiyan Liu^1,2#^, Yuanling Yu^2#^, Yadong Li^3^, Zexin Tao^4^, Lan Huang^2^, Xi Wu^5^, Yong Zhang^6,7,8^, Shuangli Zhu^6,7,8^, Qiang Sun^6,7,8^, Tianjiao Ji^6,7,8^, Dongyan Wang^6,7,8^, Ziteng Liang^1^, Shuo Liu^1,2^, Meina Cai^2^, Yimeng An^1,2^, Jierui Li^1,2^, Weijin Huang^5^, Guoyang Liao^3^, Li Yi^3*^, Lei Ma^3*^, Li Zhang^9, 4*^, Youchun Wang^1,2,3*^**

^1^Chinese Academy of Medical Sciences & Peking Union Medical College, Beijing 100730, China

^2^Changping Laboratory, Beijing 102206, China

^3^Institute of Medical Biology, Chinese Academy of Medical Sciences, Kunming 650031, Yunnan China

^4^Shandong Center for Disease Control and Prevention, Jinan 250014, China

^5^Division of HIV/AIDS and Sexually-transmitted Virus Vaccines, Institute for Biological Product Control, National Institutes for Food and Drug Control (NIFDC), Beijing 102629, China

^6^National Key Laboratory of Intelligent Tracking and Forecasting of Infectious Diseases (NITFID). National Institute for Viral Disease Control and Prevention, Chinese Center for Disease Control and Prevention, Beijing 102206, China

^7^National Polio Laboratory, World Health Organization Polio Reference Laboratory for the Western Pacific Region, National Institute for Viral Disease Control and Prevention, Chinese Center for Disease Control and Prevention, Beijing 102206, China

^8^National Health Commission Key Laboratory of Laboratory Biosafety, National Institute for Viral Disease Control and Prevention, Chinese Center for Disease Control and Prevention, Beijing 102206, China

^9^School of Public Health, Shandong University Cheeloo College of Medicine, Jinan 250012, China

^#^These authors contributed equally to this work.

^*^Correspondence: Youchun Wang (email: [wangyc@imbcams.com.cn](mailto:wangyc@imbcams.com.cn)), Li Yi (email: YL@imbcams.com.cn), Lei Ma (email: malei@imbcams.com.cn), Li Zhang (email: [ZL9127@163.com](mailto:ZL9127@163.com))

**Table S1 Titers of poliovirus pseudoviruses containing different fluorescent protein genes under different excitation wavelengths**

|  | EX 469/35 EM 525/39 | EX 531/40 EM 593/40 | EX 628/40 EM 685/40 |
| --- | --- | --- | --- |
| eGFP | **1331** |  |  |
| ZsGreen | 727 |  |  |
| YPet | 989 | 1454 |  |
| RFP |  | **1527** |  |
| mOrange |  | 373 |  |
| dTomato |  | 1521 |  |
| mCherry |  | 1431 | 1290 |
| mKate2 |  | 724 | 1937 |
| E2 |  |  | **2545** |

**
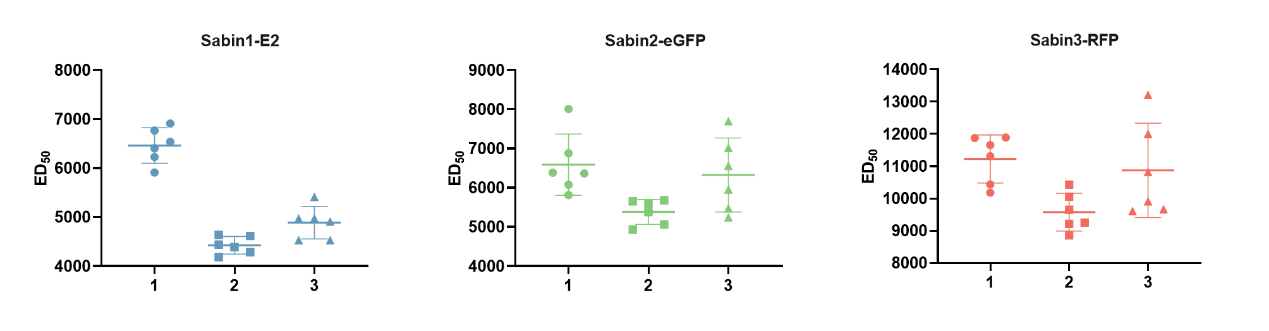
**

170mm × 45mm (300 dpi)

**Figure S1. Inter-batch consistency of pseudovirus performance.** To evaluate batch consistency of pseudovirus, we used three independently prepared batches of Sabin types 1, 2, and 3 pseudovirus to detect the neutralizing titer (ED_50_) of the same Sabin-IPV immunized rabbit serum (n=1) via the PBNA. The X-axis labels 1, 2, and 3 represent the three independent pseudovirus batches with six replicate wells per assay. The calculated inter-batch CVs were 5.01%, 9.95%, and 7.98% for types 1, 2, and 3, respectively, demonstrating high consistency among different pseudovirus batches in the assay application.

**
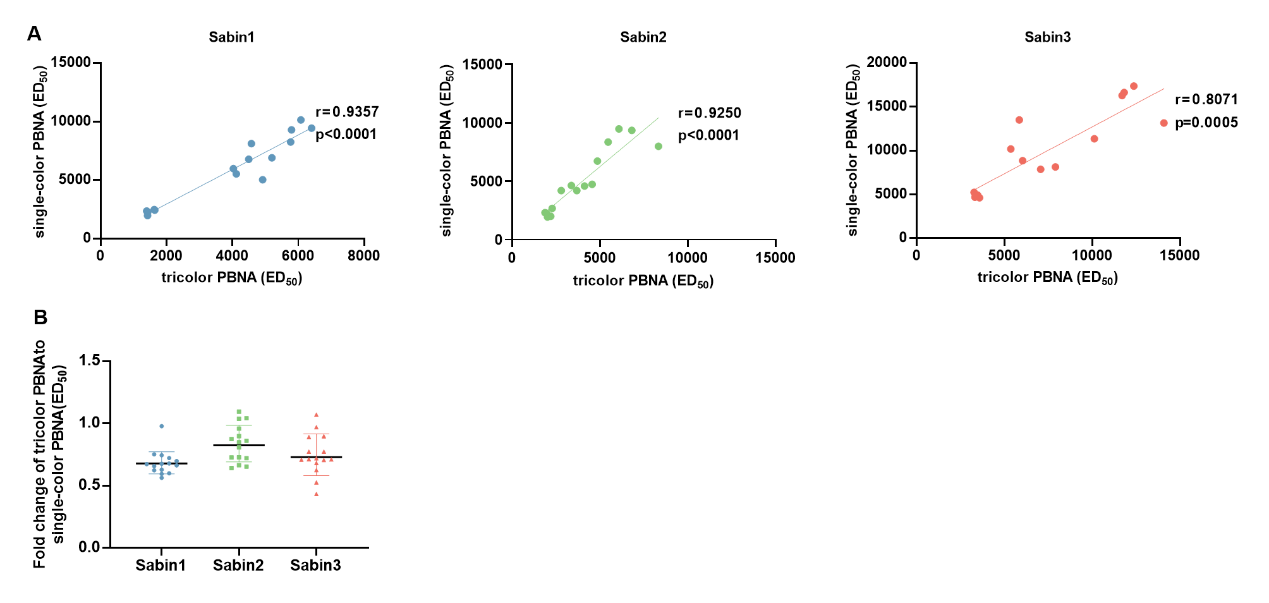
**

170mm × 81mm (300 dpi)

**Figure S2. Analysis of the consistency between** **tricolor PBNA and single-color PBNA. (A) Correlation analysis.** Neutralizing titers (ED₅₀) against poliovirus types 1, 2, and 3 from rabbit sera (n=15) were measured using both the tricolor PBNA and single-color PBNA. The ED_50_ values of two assays were then compared using Spearman correlation analysis. The trend line is represented by a solid line in the graph, r indicates the correlation between the two assays, and p<0.05 indicates a significant difference in the results. **(B) Fold-difference between** **tricolor PBNA and single-color PBNA.** The Y-axis shows the fold change, calculated as the ratio of the ED₅₀ from the tricolor PBNA to that from the single-color PBNA. The horizontal line indicates the geometric mean of the ED_50_ for different serotypes.
